# Drought stress-induced physiological and molecular changes in strawberries: an integrated transcriptomic and metabolomic perspective

**DOI:** 10.3389/fpls.2025.1679472

**Published:** 2025-12-05

**Authors:** Huimin Qiu, Tiao Ning, Huilan Ma, Weijun Gong, Diyan Li, Yanfen Niu, Zebin Chen, Lu Jin, Chengchou Han, Yilian Tang, Changjun Deng, Mingfang Zhao, Xingguo Cui, Jing Li

**Affiliations:** 1 Engineering Research Center for Urban Modern Agriculture of Higher Education in Yunnan Province, School of Agriculture and Life Sciences, Kunming University, Kunming, Yunnan, China; 2 School of Mathematics Kunming University, Kunming University, Kunming, Yunnan, China; 3 School of Pharmacy, Chengdu University, Chengdu, Sichuan, China; 4 School of Mechanical and Electrical Engineering, Kunming University, Kunming, Yunnan, China; 5 Yunnan Hanzhe Technology Co., Ltd., Kunming, Yunnan, China; 6 Yulong County Jiuhe Xinxing Agricultural Development and Planting Co., Ltd., Lijiang, Yunnan, China; 7 Shangri-La Zangmei Agricultural Technology Co., Ltd., Diqing, Yunnan, China

**Keywords:** drought stress, strawberry, transcriptomics, metabolomics, multi-omics integration

## Abstract

Strawberry (*Fragaria* × *ananassa*) is a nutritionally valuable and widely popular fruit worldwide. Drought stress is a key factor affecting strawberry production; however, previous studies lacked in depth research on the physiological, biochemical, and molecular regulatory mechanism differences among various strawberry varieties. This study systematically examined the physiological and molecular responses of two cultivars, ‘Benihoppe’ and ‘Kaorino’, to drought stress. Under mild and severe drought conditions, significant changes were observed in the growth parameters, chlorophyll concentration, antioxidant enzyme activity, and proline accumulation of the two varieties. with ‘Kaorino’ exhibiting superior drought tolerance compared with ‘Benihoppe’. Transcriptomic analysis identified 34,168 differentially expressed genes, including 9,665 upregulated and 24,503 downregulated genes. Venn analysis revealed 229 genes associated with proline biosynthesis, MDA accumulation, and antioxidant enzyme regulation. Transcription factors(TFs) expression was profiled using cross-referenced databases. A total of 8,379 DEGs encoding TFs were identified and classified into 47 TF families, some of which (e.g., NAC and WRKY) are known to be involved in drought stress responses. Kyoto Encyclopedia of Genes and Genomes (KEGG) enrichment analyses suggest that drought tolerance in strawberry involves the coordinated activation of stress signaling pathways, metabolic reprogramming, hormonal regulation, and defense-related biosynthetic routes, with both shared and cultivar-specific features. Metabolomic analysis revealed dynamic shifts in metabolites associated with osmotic adjustment, antioxidant defense, and hormonal regulation. The integrated multiomics approach enabled the construction of a gene–metabolite regulatory network, clarifying the interactions between gene expression and metabolite accumulation. Key pathways implicated in the drought response included the glycerophospholipid metabolism and MAPK signaling cascade. Lysophosphatidylglycerol acyltransferase(*LPGAT*) and Sucrose non-fermenting 1-related protein kinase 2(*SnRK2)* may be key genes affecting the drought resistance differences between two strawberry varieties. These findings provide valuable insights into the physiological and molecular mechanisms underlying drought adaptation in strawberries, offering a theoretical basis for breeding drought-resistant cultivars.

## 1 Introduction

Strawberry (*Fragaria* × *ananassa*) is a nutritionally rich and economically important fruit crop, widely valued for its distinctive flavor and health promoting properties (Wang et al., 2024). It contains diverse phytonutrients, including phenolics, polyphenols, fiber, micronutrients, and vitamins, which contribute to anti-inflammatory, anti-carcinogenic, and cardioprotective effects (Charoenwoodhipong et al., 2024). These attributer, along with their delicious taste, enhance the appeal of strawberries to consumers, thereby driving a steady increase in global demand for the fruit (R. et al., 2023).

To adapt to the rapid growth of the strawberry market, determining how to effectively enhance the yield and quality of strawberries is a crucial issue for supporting the development of the strawberry industry. The key factors influencing strawberry production include water management, nutrient application, and pollination (Teresa et al., 2021; Xue et al., 2023), among which water plays a decisive role. Strawberries exhibit low water use efficiency. For instance, blueberries require approximately 9.0 × 10^3^ m^3^ of irrigation water per hectare to achieve 60% irrigation efficiency. whereas strawberries require approximately 8.0 × 10^3^ m^3^ha^-1^ to achieve only 40% irrigation efficiency (Gavilan et al., 2024). Moreover, strawberries are highly sensitive to water scarcity. Owing to their herbaceous nature and shallow root system, drought stress can severely impair strawberry growth, yield, and quality (Li et al., 2021; Thokchom and Hazarika, 2022). This challenge is further intensified by climate change and global warming, which exacerbate production difficulties in arid and semi-arid regions (Luis et al., 2022).

Strawberry’s response to drought stress are complex, involving morphological, physiological, biochemical, and molecular adjustments aimed at survival and adaptation (Toscano et al., 2019; Gulen et al., 2021; Bistgani et al., 2024).Under drought conditions, strawberries exhibit reduced water availability, which activates multiple defense mechanisms. Physiologically, drought stress causes a decline in net photosynthetic rate, stomatal conductance, and transpiration rate as stomata close to conserve water (Toscano et al., 2019; Juan et al., 2022; Weidong et al., 2023). Although this reduces water loss, it simultaneously limits carbon dioxide uptake, leading to impaired photosynthesis. Consequently, leaf water content and turgor pressure decline, which can cause wilting and growth inhibition (Toscano et al., 2019; Sakshi et al., 2024). Biochemically, drought induces the generation of reactive oxygen species (ROS), including superoxide anions (O_2_^•−^), hydrogen peroxide (H_2_O_2_), and hydroxyl radicals (•OH) (Sharma and Zheng, 2019). Excessive ROS accumulation causes oxidative damage to DNA, amino acids, and lipids, resulting in lipid peroxidation and protein dysfunction (Sharma and Zheng, 2019; Bistgani et al., 2024). Plants counteract this by activating antioxidative enzymes such as superoxide dismutase, catalase (CAT), and peroxidase (POD), as well as accumulating osmolytes including proline, glycine-betaine, and soluble sugars to maintain osmotic balance and protect cells (Sharma and Zheng, 2019; Guohua et al., 2021). However, under severe drought, the antioxidant system is compromised, contributing to elevated ROS levels and exacerbated oxidative damage (Sharma and Zheng, 2019). For example, in *Sesuvium portulacastrum*, a halophyte, chlorophyll content decreases under salt treatment, which is physiologically similar to drought stress (Guohua et al., 2021). Molecularly, drought stress regulates the expression of genes involved in stress responses, including those associated with abscisic acid biosynthesis, osmoprotectant production, and antioxidant defense (Songquan and Yanrong, 2002; Qiang et al., 2022; Gao et al., 2024). Abscisic acid(ABA) is a key regulator of stomatal closure and stress-responsive gene activation (Sharma and Zheng, 2019; Gao et al., 2024). In wild strawberry (Fragaria nilgerrensis), integrated transcriptomic and methylome analyses have identified DNA methylation as a mechanism contributing to drought stress memory (Qiang et al., 2022; Zi et al., 2024). The FvICE1 gene in Fragaria vesca has been shown to play an essential role in cold and drought tolerance as a transcription factor (TF) (Jiaxin et al., 2023). Moreover, the heterologous expression of an *Arabidopsis thaliana* gene that can enhance auxin (Indole-3-acetic acid IAA) accumulation and root growth has been reported to improve drought tolerance in strawberry (Li et al., 2021). Drought also affects fruit quality. While severe drought reduces yield, mild drought can enhance the levels of beneficial phytochemicals (e.g., phenolics, anthocyanins, and L-ascorbic acid), accompanied by stronger antioxidant activity (Perin et al., 2019; Nafiye and Volkan, 2023). This effect is linked to ABA-mediated physiological and metabolic processes( (Perin et al., 2019). In contrast, strawberry aroma, which is mainly determined by esters, is highly vulnerable to drought, thereby reducing the overall fruit quality (Arriaza et al., 2025).

Investigating the mechanisms underlying strawberry drought responses is of scientific interest and practical relevance to production. Despite numerous studies on various plant species, in-depth analyses of strawberries remain limited, particularly regarding their molecular mechanisms. In this study, the physiological responses and molecular mechanisms of two major cultivated strawberry varieties in China under drought stress were compared through the integration of transcriptomics and metabolomics. Transcriptome analysis identified differentially expressed genes (DEGs) involved in osmotic regulation, antioxidant defense, and hormone signaling. Metabolomics analysis revealed drought-induced fluctuations in osmolytes (e.g., proline and soluble sugars), antioxidants (e.g., glutathione and flavonoids), and hormones (e.g., ABA and IAA). Combined transcriptomic and metabolomic analyses enabled the construction of a gene-metabolite regulatory network, providing preliminary insights into the interplay between gene expression and metabolite accumulation. This study advances the understanding of the physiological and molecular basis of drought response in strawberry and offers a reference for breeding drought-tolerant cultivars.

## 2 Materials and methods

### 2.1 Plant material and growth conditions

The two strawberry cultivars used in this study, ‘Benihoppe’ and ‘Kaorino’, were commercial seedlings from Yunnan Shangri-La Zangmei Agricultural Technology Co., Ltd. (Shangri-La, elevation 3,400 m). ‘Benihoppe’ is an early maturing variety originating from Japan. Its adaptability, shallow dormancy, large fruit size, and excellent quality have rendered it as an important parental line and a frequent subject of biochemical studies (Song et al., 2024). After its introduction into China, it has been widely cultivated under the names ‘Hongyan’ or ‘Red Face’. In addition, ‘Kaorino’, referred to as ‘Xiangye’ or ‘Suizhu’ in different regions of China, is extensively planted because of its strong disease resistance (Kitamura et al., 2015). However, there are no previous reports on its drought tolerance, which is the basis for its selection in comparison with ‘Benihoppe’.

After 30 d of acclimation in a smart greenhouse (50 m^2^, Kunming University, 24°58′30.98″N, 102°47′57.98″E) equipped with an automated drip-irrigation system (Netafim, 2L h^−1^ per dripper), uniform seedlings (5–6 true leaves, height 12 ± 1 cm) were selected. The growth conditions were maintained at (25 ± 3) °C/(18 ± 3)°C (day/night), relative humidity (RH) 65% ± 5%, and CO_2_400 ± 20 ppm. Plants were potted individually in 1.2 L plastic pots (10.8 cm Ø × 13.1 cm height) containing a sterilized substrate (peat: composted earthworm castings, 2:1 v/v; pH 6.0 ± 0.2, EC 0.45 dS m^−1^).

### 2.2 Drought treatment and experimental design

The soil volumetric water content (θ, 0–10 cm depth) was monitored daily at 08:00 and 18:00 using a calibrated TZS-2X sensor Field capacity (FC) was determined gravimetrically after 24 h of free drainage (θFC = 42% v/v). Three irrigation regimes were applied for 18 d:


CK:75%±5%FC(θ≈31.5%)

MD (Mild drought):55%± 5% FC(θ≈23%)

SD(Severe drought):45%± 5% FC(θ≈19%)

Each cultivar × regime combination included six biological replicates (one plant = one replicate), arranged in a randomized complete block design (total 108 pots). Pots were rotated every 3 d to minimize positional effects. θ was restored to the target levels within 30 min using automated drip irrigation controlled by a Netafim-brand PLC unit.

### 2.3 Sampling strategy

Root and fully expanded leaf samples (fourth leaf from apex) were harvested at 0, 6, 12, and 18 d after treatment initiation (DAT) between 09:00 and 10:00. Roots were gently rinsed with ice-cold 5 mM CaCl_2_, blotted dry, flash-frozen in liquid N_2_, and stored at −80°C until analysis. For each replicate, tissues from three plants were pooled to reduce individual variation while maintaining six independent replicates per treatment.

### 2.4 Physiological and biochemical assays

#### 2.4.1 Photosynthetic pigments

Leaf tissue (0.1 g) was extracted in 5 mL of 80% acetone at 4°C in the dark, and absorbance was recorded at 470, 645, and 663 nm (UV-1800, Shimadzu). Chl a, chl b, and carotenoid concentrations were calculated according to Lichtenthaler (1987).

#### 2.4.2 Malondialdehyde, proline, and antioxidant enzymes

Root or leaf tissue (0.2 g) was homogenized in 5 mL of ice-cold 50 mM phosphate buffer (pH 7.8, 1% PVP, 0.1 mM EDTA). MDA content was measured using the thiobarbituric acid method. Proline was measured using the acid-ninhydrin method, Superoxide dismutase (SOD) (NBT inhibition), POD (guaiacol oxidation), and CAT (H_2_O_2_ decomposition) activities were determined using commercial kits (Solarbio, Beijing; catalog numbers BC0020, BC0290, BC0170, BC0090, and BC0200) according to the manufacturer’s microplate protocols. Absorbance was recorded using a SpectraMax iD3 (Molecular Devices). Enzyme activities were expressed as U mg^−1^ protein, with soluble protein quantified using the Bradford assay (BSA as a standard).

### 2.5 RNA extraction and library construction

Total RNA was extracted from 100 mg of root tissue using TRIzol^®^ Reagent (Invitrogen, 15596026) according the manufacturer’s instructions and purified using DNase I (Takara, 2270A). RNA integrity was verified on 1.2% agarose gels and with an Agilent 2100 Bioanalyzer (RIN ≥ 8.0). The concentration was quantified using a Qubit 4.0 fluorometer (Invitrogen). Only high-quality RNA sample [OD260/280 = 1.8~2.2,OD260/230≥2.0,RQN≥6.5, 28S:18S≥1.0, >1μgwas used to construct sequencing library. Strand-specific libraries were prepared using the NEBNext Ultra RNA Library Prep Kit for Illumina (NEB, E7770) following the 1 µg low-input protocol (insert size 300 ± 50 bp). Unique dual-index adapters (NEB, E7600) were ligated, and the libraries were pooled equimolarly.

### 2.6 RNA sequencing and data processing

Paired-end 150bp sequencing was performed on an Illumina NovaSeq 6000 platform (Illumina, San Diego, CA, USA) at Shanghai Majorbio Bio-Pharm Technology Co., Ltd, yielding at least 6 Gb of clean data per sample. Raw reads were adapter and quality trimmed using Trimmomatic v0.39 (SLIDINGWINDOW:4:20, MINLEN:50). Clean reads were aligned to the *Fragaria vesca* reference genome v4.0 (https://www.rosaceae.org) using HISAT2 v2.2.1 (–dta, –rna-strandness RF). Gene-level counts were generated using FeatureCounts v2.0.1 (annotation: *F. vesca* v4.0.a2). Differential expression was analyzed in DESeq2 v1.34.0; genes with |log_2_ fold-change| ≥ 1 and FDR-adjusted *P* < 0.05 were defined as DEGs. Gene Ontology (GO) and KEGG enrichment analyses were performed using clusterProfiler v4.2.2 with a q-value threshold of 0.05.

### 2.7 Metabolite extraction and UHPLC-HRMS analysis

Metabolites were extracted from 50 mg of finely ground root powder with 1 mL of 80% methanol (pre-cooled at −20°C, containing 0.1 mg mL^−1^ lidocaine as an internal standard). Following vortexing (30s) and sonication (10 min, 4°C), the samples were centrifuged at 12,000 × g for10 min at 4°C). Supernatants were filtered through 0.22 µm PVDF membranes and analyzed on a Vanquish UHPLC system coupled to a Q Exactive HF-X Orbitrap MS (Thermo Fisher). Chromatographic separation was performed on a Hypersil GOLD C18 column (2.1 × 100 mm, 1.9 µm) at 40 °C. The mobile phases consisted of 0.1% formic acid in water (A) and acetonitrile (B), with the following gradient: 0–1 min, 5% B; 1–9 min, 5-95% B; 9–11 min, 95% B; 11-11.1 min, 95-5% B; 11.1–14 min, 5% B. The flow rate was 0.3 mL min^−1^. MS parameters included a full-scan range of 70-1,050 *m/z*, resolution of 120,000, spray voltages of +3.5/−2.8 kv, capillary temperature of 320 °C, and probe heater temperature of 350°C. Raw files were processed using Compound Discoverer 3.3 (Thermo) with a 5 ppm mass tolerance against HMDB, and METLIN and an in-house *Fragaria* spectral library. Peak areas were normalized to the internal standard and fresh weight. Differential metabolites were identified by OPLS-DA (SIMCA 16.0) with VIP > 1 and *P* < 0.05 (Student’s *t*-test, FDR-adjusted).

### 2.8 Integration of transcriptomic and metabolomic data

Pearson correlation coefficients were calculated between normalized metabolite abundances (log_2_- transformed) and DESeq2-normalized gene counts for each treatment × time combination. Gene-metabolite pairs with |*r*| ≥ 0.8 and *P* < 0.01 were retained. Pathway mapping was performed using KEGG Mapper v4.0, with *F. vesca* as the reference.

### 2.9 Statistical analyses

Physiological and biochemical data were presented as mean ± SD (*n* = 6). Normality (Shapiro-Wilk test) and homogeneity of variances (Levene test) were verified prior to one-way ANOVA. Significant differences among treatments were separated by Tukey’s HSD test at *P* < 0.05 using SPSS v26.0. Figures were prepared using GraphPad Prism v9.0 and Adobe Illustrator v26.0.

## 3 Results

### 3.1 Effects of drought stress on the morphology, physiology, and biochemistry of strawberries

**Figure 1:**
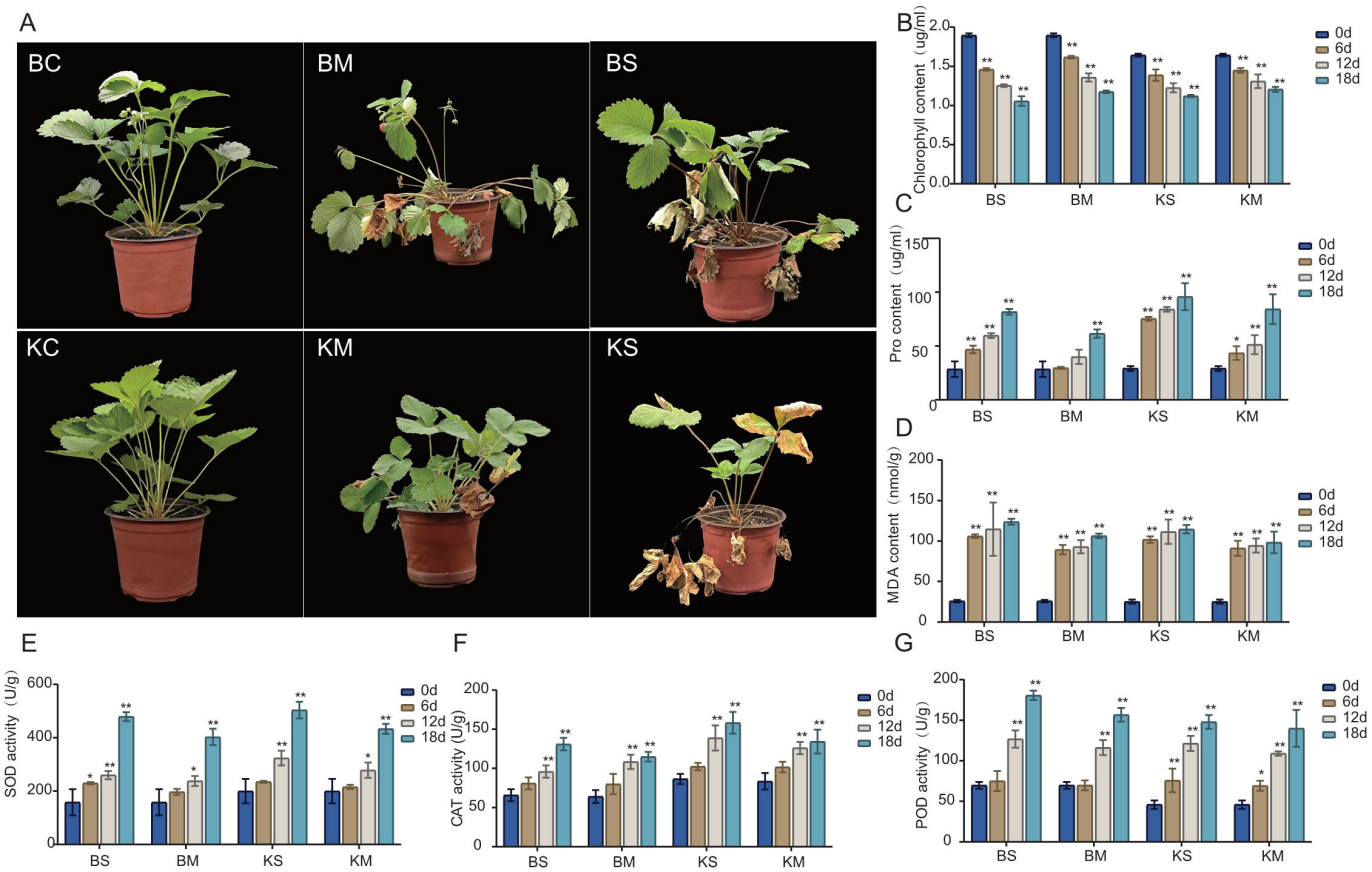
Effects of drought stress on strawberry physiology and morphology. **(A)** Phenotypic responses of strawberry plants under control, mild drought, and severe drought stress treatments; (BC: Benihoppe Root Control;BM: Benihoppe Root Mild Drought;BS;Benihoppe severe drought stress. KC, Kaorino Root Control; KM, Kaorino Root Mild Drought; KS, Kaorino Root Severe Dtought). **(B–G)** Changes in physiological and biochemical indices, including chlorophyll content, proline accumulation, and antioxidant enzyme activities, measured at different time points. Asterisks denote statistical significance: *P* < 0.05 (*), *P* < 0.01 (**).

The physiological and biochemical responses of two strawberry cultivars, (‘Benihoppe’ and ‘Kaorino’) were evaluated under drought stress. The treatments induced significant alterations in growth parameters, chlorophyll concentration, antioxidant enzyme activities, and proline accumulation in both cultivars. Marked morphological changes were observed in both cultivars under varying drought stress conditions. In the control group (CK), plants exhibited overall vigor, characterized by green leaves and robust growth (**Figure 1A–BC, KC**). Under mild drought stress, obvious leaf yellowing and partial withering occurred, leading to inhibited growth and a damaged state (**Figure 1A–BM, KM**). Under severe drought stress, leaf yellowing became widespread, and numerous leaves withered, indicating severe plant damage (**Figure 1A–BS, KS**). Overall, increasing drought severity resulted in a gradual transition of leaves from green to yellow, accompanied by growth inhibition and severe wilting. Under varying drought stress conditions, the chlorophyll concentration significantly decreased in both cultivars as drought stress intensified. Within each cultivar, reductions were greater under severe stress than under mild stress. Comparative analysis revealed that ‘Benihoppe’ initially exhibited higher chlorophyll content than ‘Kaorino’. However, under identical drought conditions, the reduction in chlorophyll was more pronounced in ‘Benihoppe’ than in ‘Kaorino’(**Figure 1B**). Proline levels significantly increased in both cultivars under drought stress. Within the same cultivar, the increase were greater under severe stress than under mild stress. The initial proline contents were similar between the cultivars, but under identical drought conditions, the increase in ‘Kaorino’ was significantly higher than that in ‘Benihoppe’ (**Figure 1C**). The baseline MDA was essentially identical in both cultivars, and both increased with increasing drought severity. However, the increase in MDA was slightly greater in ‘Benihoppe’ than in ‘Kaorino’(**Figure 1D**). For antioxidant enzyme activities, both cultivars exhibited upward trends in SOD, POD, and CAT with increasing drought severity. The increases in SOD and CAT activities were less pronounced in ‘Benihoppe’ than in ‘Kaorino’, whereas POD activity exhibited a more substantial increase in ‘Benihoppe’ than in ‘Kaorino’ (**Figure 1E–G**).

### 3.2 Transcriptome sequencing results

In ‘Benihoppe’, the BC, BM, and BS groups generated 46.16 million, 44.70 million, and 43.40 million raw reads, respectively. Similarly, in ‘Kaorino’, the KC, KM, and KS groups produced 45.30 million, 40.67 million, and 44.14 million raw reads, respectively. Quality assessment confirmed that Q20 and Q30 values exceeded 95% in all libraries, indicating that the sequencing data satisfied the requirements for downstream bioinformatics analyses (**Supplementary Table S1**). Based on these findings, DEG analysis was performed to evaluate transcriptional changes under mild and severe drought conditions in both cultivars.

### 3.3 Differentially expressed genes under drought stress

**Figure 2:**
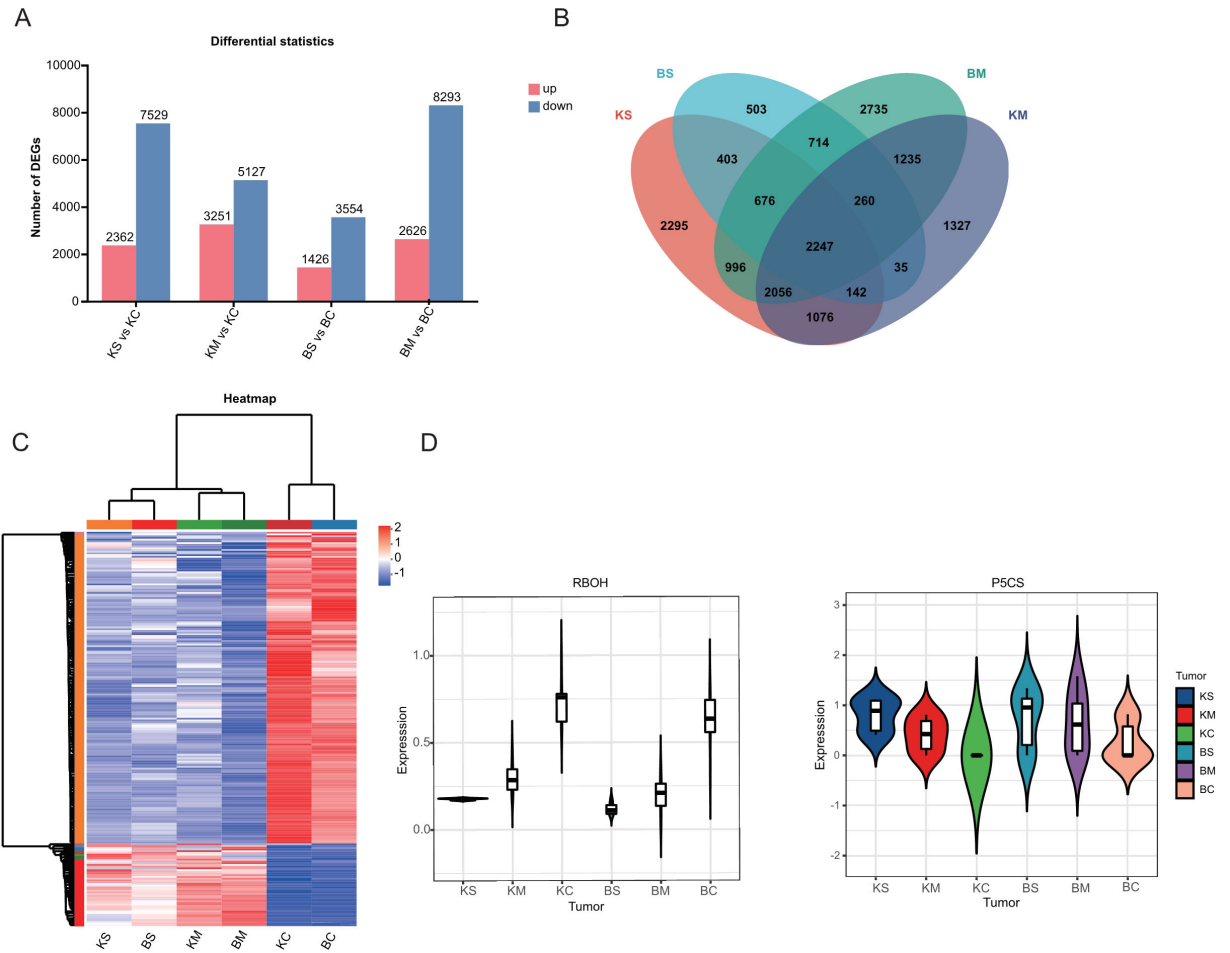
Identification and transcriptome analysis of DEGs. **(A)** Number of DEGs (upregulated and downregulated) under mild and severe drought stress. **(B)** Venn diagram showing shared and unique DEGs across drought treatments within each cultivar. **(C)** Heatmap displaying expression patterns of DEGs under various drought conditions. **(D)** Violin plots depicting expression levels of key drought-responsive genes (RBOH, P5CS).

To investigate the gene regulatory responses in the two strawberry cultivars, transcriptome analysis of root tissues under drought stress identified 34,168 DEGs, including 9,665 upregulated and 24,503 downregulated genes Across the four comparison groups, including BS vs. BC, BM vs. BC, KS vs. KC, and KM vs. KC, 4,980, 10,919, 9,891, and 8,378 DEGs were detected, with 1,426, 2,626, 2,362, and 3,251 genes upregulated, and 3,554, 8,293, 7,529, and 5,127 genes downregulated, respectively (**Figure 2A**). The predominance of downregulated DEGs suggests a potential adaptive strategy under drought stress involving the suppression of energy-intensive metabolic processes, growth modulation and activation of defense pathways. Venn diagram analysis (**Figure 2B**) identified 3,897 DEGs shared between mild and severe drought treatments in ‘Benihoppe’ (2,735 mild-specific and 503 severe-specific),and 5,521 in ‘Kaorino’ (1,327 mild-specific, and 2,295 severe-specific). A total of 2,247 DEGs were commonly regulated in both cultivars, indicating conserved transcriptional responses to drought stress. These findings highlight transcriptional suppression as a central mechanism of resource reallocation and stress adaptation. Venn analysis of the 2,247 shared DEGs (**Figure 2C**) revealed 229 genes associated with proline biosynthesis, MDA accumulation, and antioxidant enzyme regulation. Cluster heatmap analysis indicated consistent expression trends in both cultivars. Notably, the expression of the respiratory burst oxidase homolog (RBOH) gene increased significantly with drought severity (**Figure 2D**), consistent with its role in ROS-mediated signaling and oxidative stress defense (Nour et al., 2024). Similarly, the gene encoding Δ^1^-pyrroline-5-carboxylate synthase (P5CS), a key enzyme in proline biosynthesis, was significantly upregulated in both cultivars, consistent with enhanced proline accumulation under drought stress (**Figure 2D**) (Farooq et al., 2009).

### 3.4 Gene ontology enrichment analysis in response to drought stress

**Figure 3:**
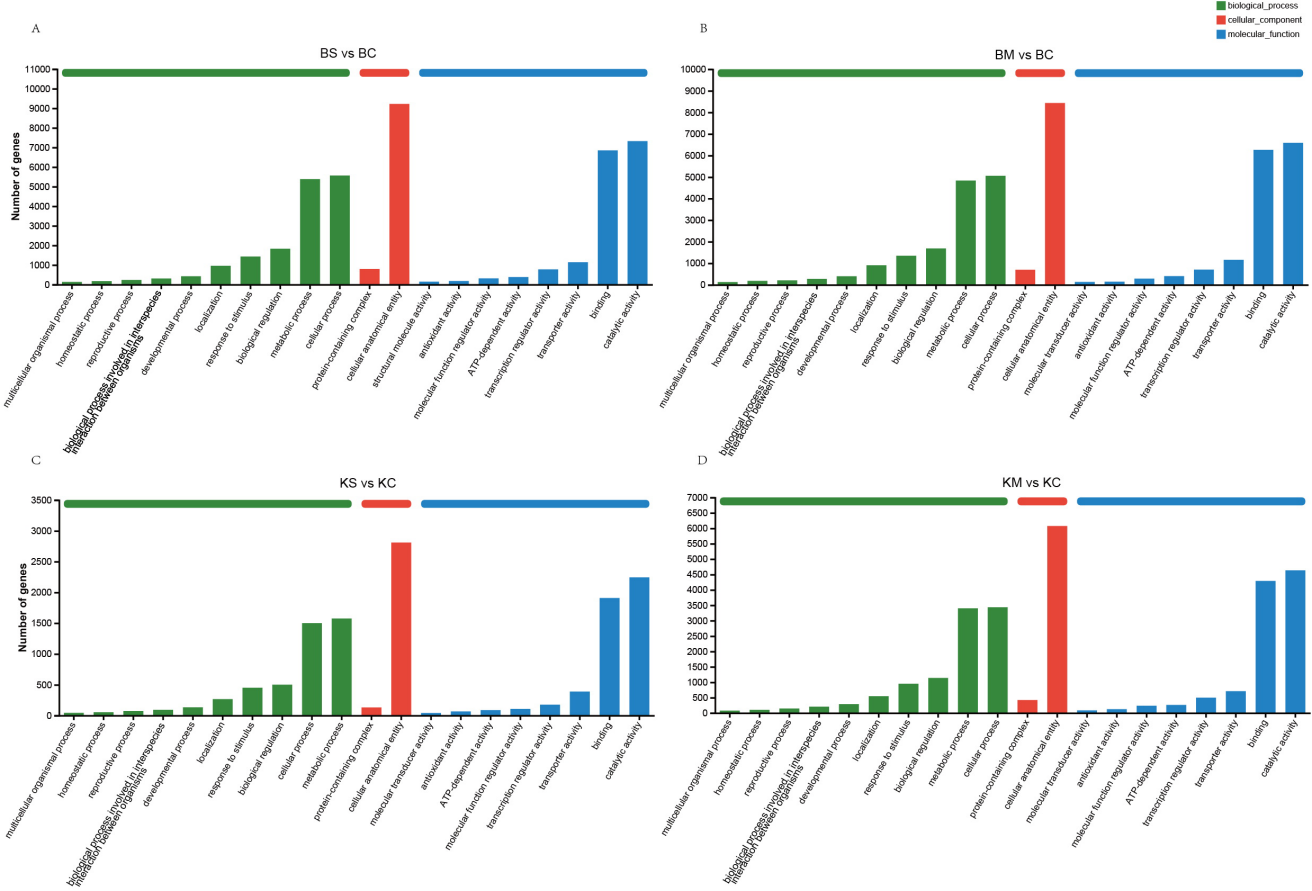
Distribution and GO enrichment analysis of DEGs under different degrees of drought stress.

GO enrichment analysis was conducted to functionally characterize the DEGs identified under different drought stress conditions. GO terms were classified into three categories: biological process (BP), cellular component (CC), and molecular function (MF). Both ‘Benihoppe’ and ‘Kaorino’ exhibited similar GO enrichment patterns under mild and severe drought stress. In the BP category, DEGs were predominantly enriched in “cellular process”, “metabolic process”, and “response to stimulus”; In the CC category, “cellular anatomical entity” and “protein-containing complex” were the most represented. In the MF category, “catalytic activity” and “transporter activity” were significantly enriched (**Figure 3**). These results indicated that both cultivars activate conserved defense pathways in response to drought. Enhanced metabolic activity (BP) and catalytic function (MF) facilitate osmolytes (e.g., proline) and antioxidant biosynthesis to counteract oxidative damage. The enrichment of transporter activity (MF) supports ion homeostasis and solute transport, whereas the reorganization of protein complexes (CC) may reflect signal transduction mediated by receptor kinases and transcriptional regulators. The consistent enrichment of “response to stimulus” across different drought intensities suggests sustained stress perception and signaling. This may reflect a systemic shift from growth processes to protective mechanisms, including cell wall remodeling and metabolic reprogramming, which enhances drought tolerance.

### 3.5 KEGG pathway enrichment analysis of DEGs under drought stress

**Figure 4:**
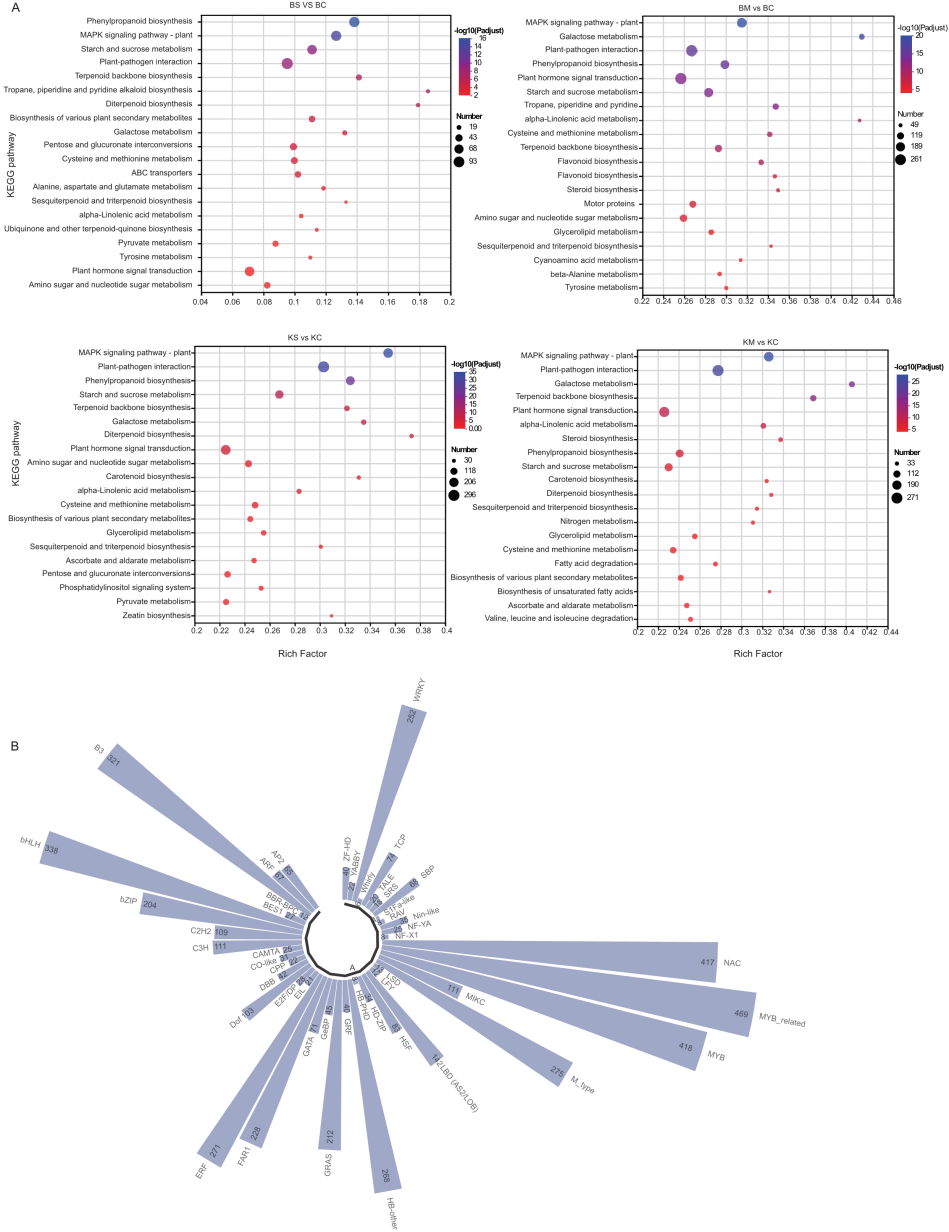
KEGG and TFs analysis of DEGs under drought stress. **(A)** KEGG pathway enrichment analysis of DEGs under different levels of drought stress; **(B)** Classification and distribution of differentially expressed TFs across treatments.

To identify the major signaling and metabolic pathways underlying drought responses, KEGG pathway enrichment analysis was performed using DEGs from four comparisons: BS vs. BC, BM vs. BC, KS vs. KC, and KM vs. KC (**Figure 4A**). Commonly enriched pathways included plant-pathogen interaction (map04626), phenylpropanoid biosynthesis (map00940), and MAPK signaling pathway-plant (map04016), all of which are integral to stress perception, signaling cascades, and defense activation. Additionally, starch and sucrose metabolism (map00500) and terpenoid backbone biosynthesis (map00900) were significantly enriched, suggesting their roles in osmotic adjustment, energy redistribution, and secondary metabolite biosynthesis under drought conditions. In particular, plant hormone signal transduction (map04075) was significantly enriched in both ‘Benihoppe’ drought treatments (BS vs. BC and BM vs. BC), implying a prominent role for hormonal regulation, particularly ABA, IAA, and ethylene, in mediating drought responses. In contrast, diterpenoid biosynthesis (map00904), associated with secondary metabolites, was highly enriched in the KM vs. KC comparison, suggesting cultivar-specific activation of specialized pathways under severe drought. Collectively, these findings suggest that drought tolerance in strawberry involves the coordinated activation of stress signaling pathways, metabolic reprogramming, hormonal regulation, and defense-related biosynthetic routes, with both shared and cultivar-specific features.

### 3.6 Analysis of differentially expressed transcription factors

TFs are key regulators of plant responses to drought stress, orchestrating complex molecular networks that enhance survival under water deficient conditions. To elucidate the regulatory mechanisms underlying drought responses in the two strawberry cultivars, TF expression was profiled using cross-referenced databases. A total of 8,379 DEGs encoding TFs were identified and classified into 47 TF families (**Figure 4B**). The top 10 TFs families by DEGs count were: MYB-related (n = 469), MYB (n = 418), NAC (n = 417), bHLH (n = 338), B3 (n = 321), M-type (n = 275), ERF (n = 271), HB-other (n = 268), WRKY (n = 252), and FAR1 (n = 228). These TFs families are associated with diverse biological functions, including stress perception, hormone signaling, secondary metabolite biosynthesis, and oxidative stress mitigation. The observed transcriptional changes emphasize the involvement of complex regulatory networks in cultivar-specific drought adaptation.

### 3.7 Identification of differentially accumulated metabolites under drought stress

**Figure 5:**
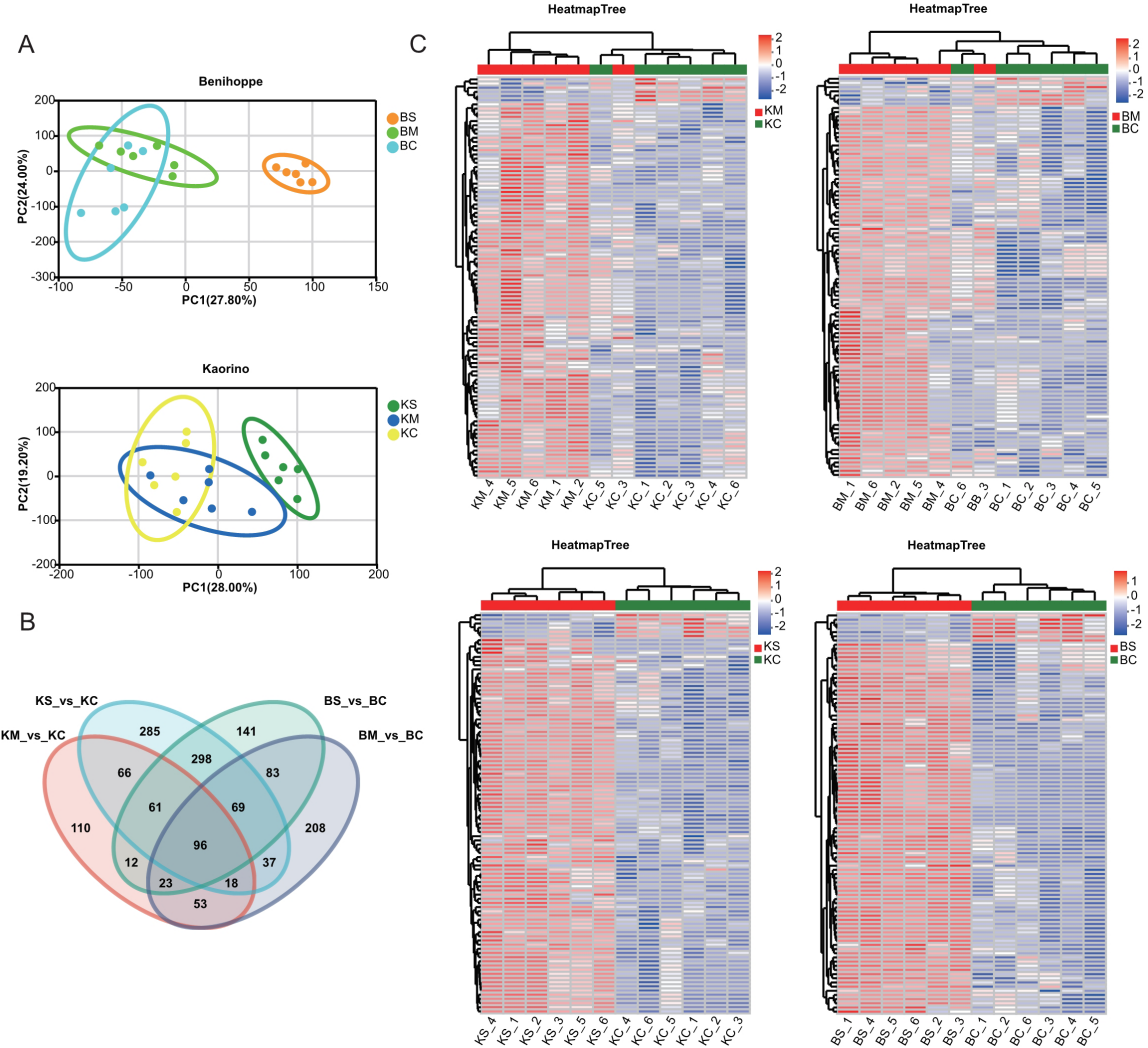
Extensive targeted metabolomic analyses of strawberry under different drought stress levels. **(A)** PCA showing sample separation under control, mild, and severe drought conditions; **(B)** Venn diagram of DAMs across drought treatments; **(C)** Heatmap clustering of DAMs compared to the control. Red and green represent high and low metabolite abundance, respectively.

To characterize metabolite-level responses to drought stress, comprehensive targeted metabolomic profiling was performed using UPLC-MS/MS. Orthogonal partial least squares-discriminant analysis (OPLS-DA) revealed clear metabolic distinctions between control and drought-stressed groups, confirming analytical reproducibility and method robustness (**Figure 5A**). Differentially accumulated metabolites (DAMs) were identified across stress gradients, and Venn diagram analysis revealed treatment and cultivar-specific metabolite profiles (**Figure 5B**). A subset of 96 DAMs was conserved across conditions, including key lipids and their derivatives—such as glycerophosphocholine and LysoPE (0:0/18:3(9Z,12Z,15Z))—and terpenoids like annuolide C. Comparative analysis revealed cultivar-specific metabolic responses. The ‘Kaorino’ cultivar exhibited 439 and 930 DAMs under mild and severe drought, respectively. In contrast, the ‘Benihoppe’ cultivar displayed 587 and 783 DAMs under the same stress conditions (**Figure 5C**). These findings suggest that metabolic reprogramming in response to drought is both stress intensity dependent and genotype specific, involving lipid remodeling, osmolyte accumulation, and secondary metabolite biosynthesis.

### 3.8 KEGG enrichment analysis of differentially accumulated metabolites

**Figure 6:**
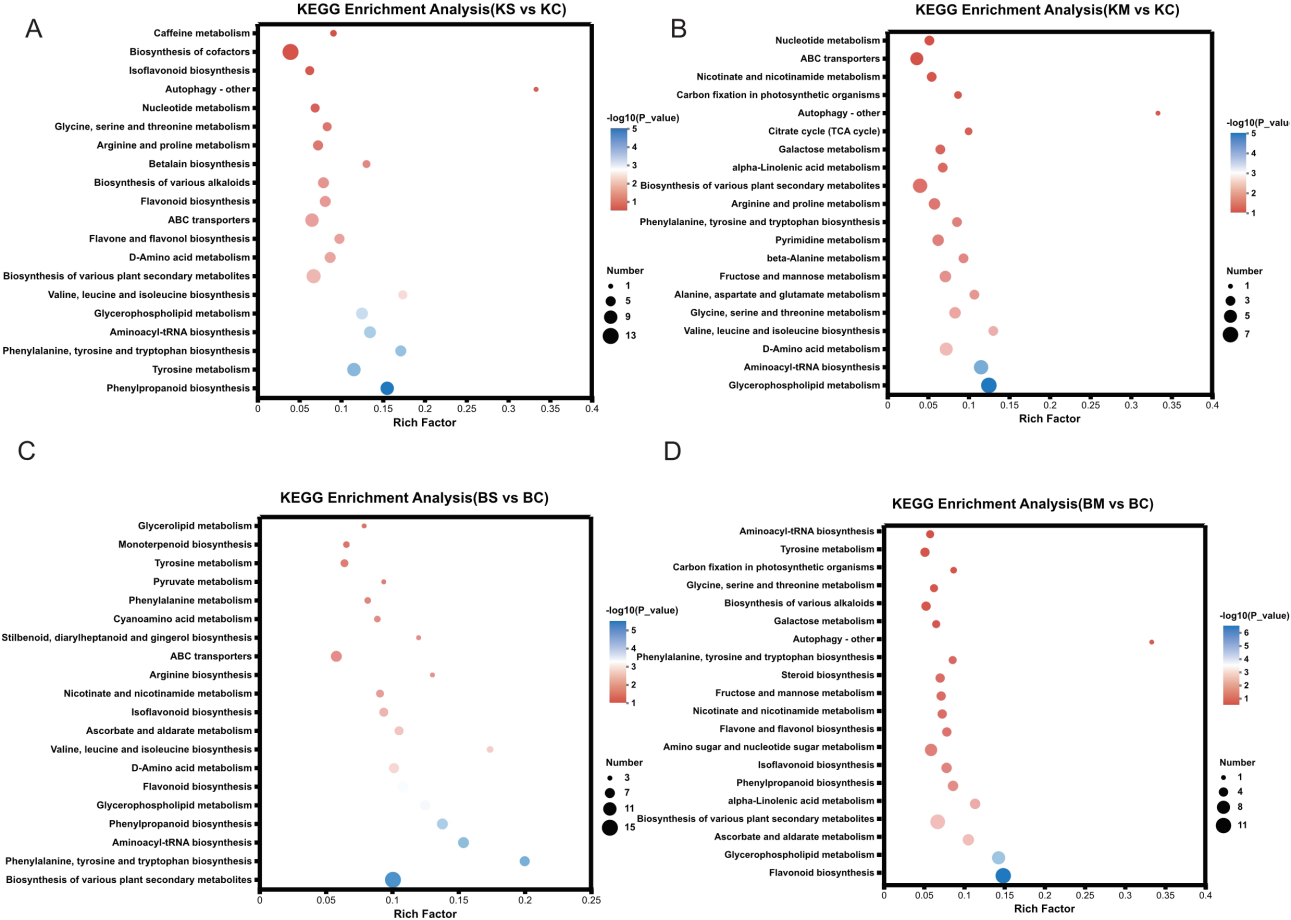
KEGG enrichment analysis of differentially accumulated metabolites.

To determine the metabolic pathways affected by drought stress, KEGG enrichment analysis was performed based on the number of significantly enriched metabolites in each treatment group. In ‘Benihoppe’, flavonoid biosynthesis and glycerophospholipid metabolism were significantly enriched in the BM vs. BC comparison, whereas the biosynthesis of various plant secondary metabolites and phenylalanine, tyrosine, and tryptophan biosynthesis were enriched in the BS vs. BC comparison (**Figure 6A–B**). In ‘Kaorino’, glycerophospholipid metabolism, aminoacyl tRNA biosynthesis, and D amino acid metabolism were enriched in KM vs KC, whereas phenylpropanoid biosynthesis and tyrosine metabolism were enriched in KS vs. KC (**Figure 6C–D**). These results demonstrate that drought stress induces both common and cultivar-specific changes in key metabolic pathways, particularly those involved in secondary metabolism, membrane lipid remodeling, and amino acid metabolism, thereby contributing to drought adaptation.

### 3.9 Integrated analysis of DEGs and DAMs

**Figure 7:**
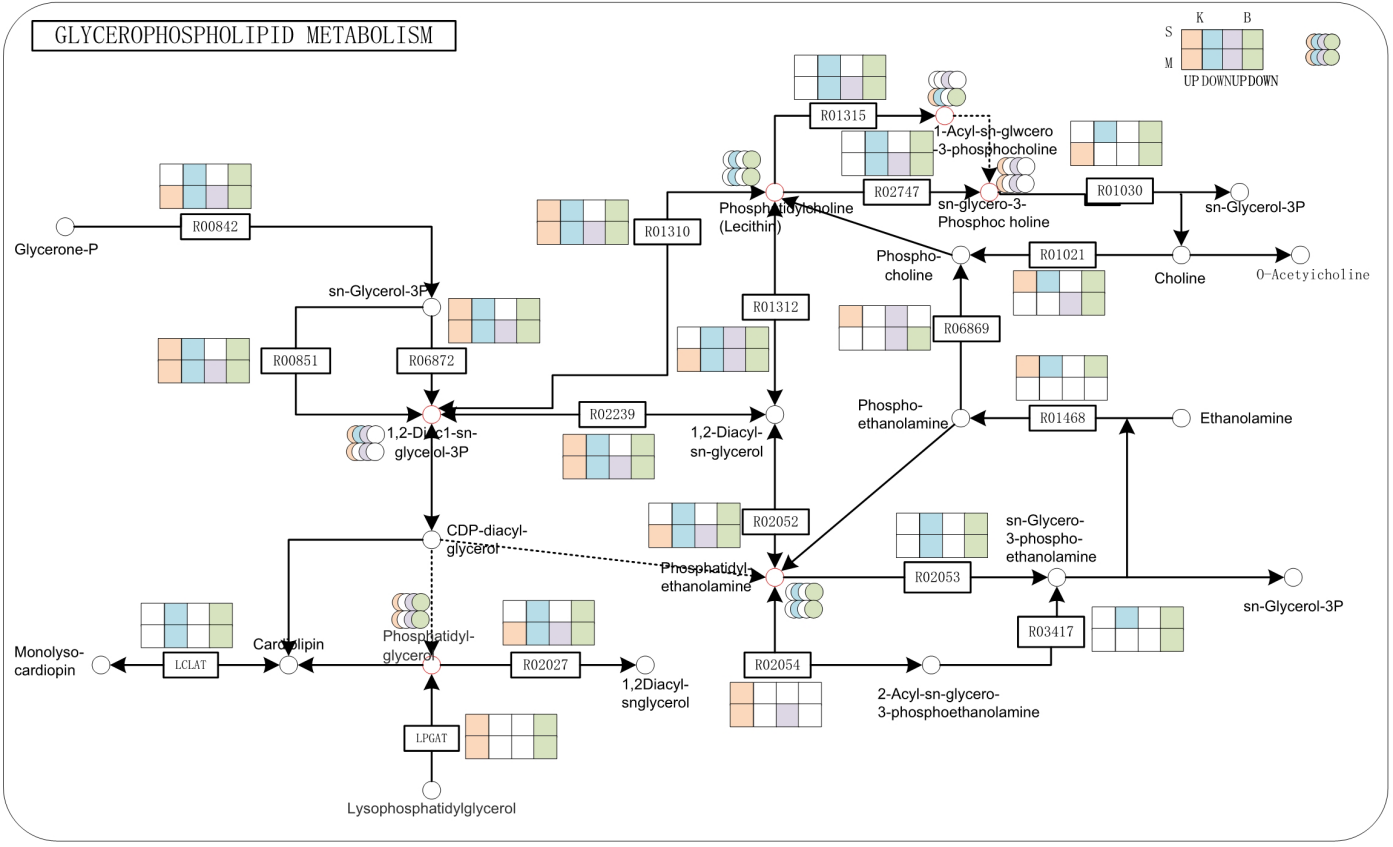
Glycerophospholipid metabolism pathway under moderate and severe drought in ‘Kaorino’. Red and purple circles represent up-regulated metabolites; green and blue circles represent down-regulated metabolites; red and purple rectangles indicate up-regulated genes; green and blue rectangles indicate down-regulated genes; B is for Benihoppe, K is for Kaorino, M is for Mild drought, S is for Severe drought.

To identify the key regulatory components contributing to drought tolerance, we focused on pathways jointly enriched with DEGs and DAMs. Two major pathways emerged: glycerophospholipid metabolism and MAPK signaling pathway. In the glycerophospholipid metabolism pathway, significant transcriptional and metabolic changes were observed under drought stress (**Figure 7**). Phosphatidylglycerol(PG) biosynthesis genes were upregulated in both cultivars, whereas genes involved in phosphatidylcholine(PC) and phosphatidylethanolamine(PE) synthesis were downregulated. The expression of *LPGAT* genes varied between cultivars, being upregulated in ‘Kaorino’ and downregulated in ‘Benihoppe’, indicating genotype-specific regulation of membrane lipid remodeling. Furthermore, intermediate metabolites such as 1,2-diacyl-sn-glycerol-3-phosphate (1,2-DiAc1-sn-glycerol-3P) and sn-glycero-3-phosphocholine accumulated significantly under drought conditions. In contrast, the expression of genes such as Fx84Dg00479 (R01315), Fx94Bg00579 (R02053), and Fxa2Cg01240 (R02747), which encode enzymes responsible for PC and PE degradation, was downregulated. This repression may limit membrane lipid degradation, thereby enhancing structural stability during drought stress.

**Figure 8:**
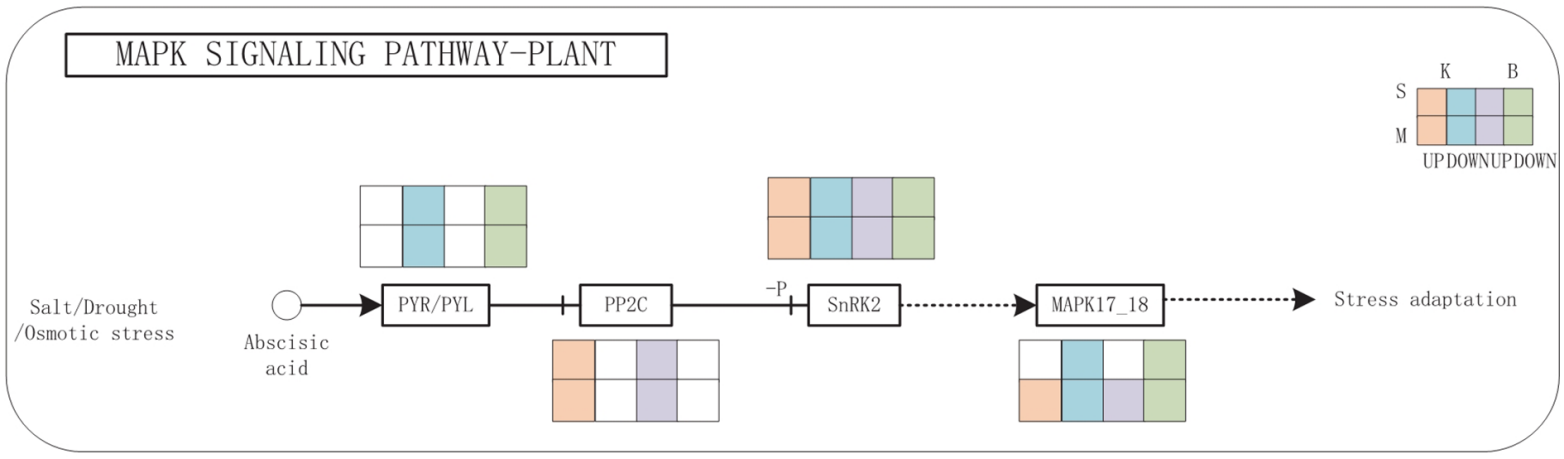
MAPK signaling pathways under drought stress. Red and purple circles represent up-regulated metabolites; green and blue circles represent down-regulated metabolites; red and purple rectangles indicate up-regulated genes; green and blue rectangles indicate down-regulated genes; B is for Benihoppe, K is for Kaorino, M is for Mild drought, S is for Severe drought.

The MAPK signaling pathway exhibited complex regulatory dynamics in response to drought stress (**Figure 8**). The expression of PYR/PYL receptor genes was downregulated, while PP2C phosphatase genes were upregulated, suggesting a negative feedback loop under prolonged stress. Interestingly, *SnRK2* kinase genes displayed a mixed expression profile, with both upregulated and downregulated isoforms depending on the cultivar and drought severity, implying nuanced regulation of ABA signal transduction. Together, these findings from integrative transcriptomic and metabolomic analyses highlight glycerophospholipid metabolism and MAPK signaling as key pathways involved in drought tolerance. The observed cultivar-specific expression patterns further suggest that differential regulation of these pathways contributes to the contrasting drought resistance observed in ‘Benihoppe’ and ‘Kaorino’.

## 4 Discussion

### 4.1 Physiological and biochemical responses to drought stress

Under varying drought stress intensities and durations, both cultivars exhibited significant morphological changes, including leaf yellowing, wilting, and withering, which are typical response of strawberries to drought conditions (Suresh et al., 2023). When evaluated solely based on morphological traits under identical conditions, ‘Benihoppe’ appeared to suffer less damage than ‘Kaorino’. However, further morphological analyses are required to determine the statistical significance of this difference.

Drought stress commonly reduces photosynthesis and transpiration rates because of the close correlation between chlorophyll content and the photosynthetic activity. Consistent with this process, chlorophyll levels decrease as a result of reduced precursor synthesis and enhanced degradation (Bistgani et al., 2024). The extent of chlorophyll decline varies across cultivars, with resistant varieties exhibiting a less pronounced reduction (Farooq et al., 2009). Under mild and severe drought stress, the chlorophyll loss in the chloroplasts of ‘Kaorino’ was less than that of ‘Benihoppe’, demonstrating a more effective chloroplast protection mechanism and stronger drought resistance in ‘Kaorino’.

Excessive drought stress can cause overproduction of ROS in plants (Sheng et al., 2022). The imbalance between ROS generation and antioxidant defense systems induces oxidative damage to cellular components, with lipid peroxidation of membranes serving as a major consequence (Yini et al., 2021). As a byproduct of polyunsaturated fatty acid peroxidation in membranes, MDA is widely regarded as a reliable marker of oxidative damage (Vardan et al., 2022). Under drought stress, the MDA levels in both cultivars increased significantly, with a greater increase in ‘Benihoppe’ than in ‘Kaorino’, indicating more severe oxidative damage in ‘Benihoppe’.

To counteract ROS, plants activate enzymatic antioxidant systems involving SOD, CAT, and POD (Hui et al., 2023). These enzymes function synergistically to detoxify ROS and maintain redox homeostasis under stress conditions (Vieira et al., 2024). SOD is the primary defense against ROS, as it converts superoxide radicals (O_2_^−^) into hydrogen peroxide (H_2_O_2_) and molecular oxygen (O_2_) (Qin et al., 2024). Because O_2_^−^ is highly reactive and capable of initiating chain oxidative reactions (Yu et al., 2024), SOD activity generally increases as a direct response to elevated O_2_^−^ production (Li et al., 2024). CAT decomposes H_2_O_2_ into H_2_O and O_2_ (Lin et al., 2024), mitigating the harmful effects of H_2_O_2_, which can cause oxidative damage if not efficiently scavenged (Parveen et al., 2019). Under drought conditions, CAT activity typically increases to cope with elevated H_2_O_2_ levels (Shen et al., 2024). POD is a broad group of enzymes that catalyze the oxidation of diverse substrates in H_2_O_2_ (Cheng et al., 2024). While SOD converts O_2_^−^ into H_2_O_2_ and CAT subsequently decomposes H_2_O_2_ into H_2_O and O_2_, POD contributes to H_2_O_2_ detoxification and frequently plays a more prominent role in cell wall stiffening and lignification, processes essential for maintaining structural integrity under stress (Huangying et al., 2024). Drought stress is generally accompanied by an increase in POD activity (Khan et al., 2025).

Proline, an osmoprotective amino acid, is another critical marker of drought stress resilience in plants. Its accumulation mitigates osmotic imbalance, stabilizes cellular structures, and scavenges ROS under water-deficient conditions (Pál et al., 2018). In the present study, the proline levels increased in both cultivars with intensified drought, whereas the increase in ‘Benihoppe’ was less pronounced than that in ‘Kaorino’, suggesting that the drought resistance of ‘Benihoppe’s’ was weaker than that of ‘Kaorino’.

In summary, ‘Kaorino’ exhibited superior drought tolerance, characterized by higher chlorophyll retention, stronger antioxidant defenses, and greater proline accumulation. These physiological and biochemical traits indicate that inherent genetic differences contribute to more robust drought adaptation mechanisms in ‘Kaorino’.

### 4.2 Integration of transcriptomic and metabolomic insights: multi-pathway regulation of drought response

Differential expression analysis identified 47 TF families that responded to drought stress in both cultivars. Key families included MYB-related, MYB, NAC, bHLH, B3, WRKY, and FAR1, several of which are known to regulate stress responses in other plant species.

In this study, both NAC and WRKY families showed significant expression changes under drought stress, suggesting that strawberries activate these TFs to modulate growth, development, and defense processes. The upregulation of NAC genes may promote adaptive developmental changes, whereas WRKY expression changes likely contribute to enhanced defense signaling. Furthermore, the NAC family is a master regulator of stress in plants. NAC TFs bind to cis-elements (such as NACR) to activate genes involved in osmoprotectant synthesis, ROS clearance, and stomatal closure (Abdul et al., 2024; Zhang et al., 2024). The WRKY family is ABA-dependent, and WRKY TFs can modulate ABA biosynthesis and stomatal aperture via MAPK cascades (Kiranmai et al., 2018; Dingyue et al., 2023).Overexpression of MdWRKY56 in apple increased proline accumulation (2.5-fold) and reduced water loss by 30% via NCED3 (an ABA biosynthesis gene) activation (Dingyue et al., 2023). MYB is involved in metabolic reprogramming, with MYB TFs (e.g., MYB96) redirecting carbon toward triacylglycerol synthesis, thereby conserving energy during stress (Lee et al., 2019).

Our integrated analysis revealed that strawberries coordinate multiple signaling and metabolic pathways under drought stress, particularly the glycerophospholipid metabolism and MAPK signaling cascade —each contributing uniquely to drought tolerance. Glycerophospholipids, as major membrane constituents, are critical for maintaining membrane structure and signaling under stress (Matsuo et al., 1992). In our study, drought stress altered lipid profiles: PG levels increased, while PC and PE levels decreased. Genes promoting PG synthesis were upregulated, indicating active reinforcement of membrane integrity. In contrast, genes such as Fx84Dg00479 and Fx94Bg00579, involved in PC and PE degradation, were downregulated, suggesting suppressed membrane breakdown. Interestingly, expression of *LPGAT*, a gene involved in PG biosynthesis, was cultivar-dependent: upregulated in ‘Kaorino’ and downregulated in ‘Benihoppe’. These findings align with previous studies showing PG accumulation in stress-tolerant species. For example, Increased PG content in chloroplast membranes of cold-tolerant plants (Matsuo et al., 1992, and PG species critical for photosynthetic stability in salt-tolerant Dunaliella (Lynch and Thompson, 1984). MAPK signaling mediates environmental stress perception and signal transduction. The MAPK gene exhibited differential expression between mild and severe drought, showing upregulation under moderate stress and downregulation under severe stress. ABA is a core regulator of plant drought responses. Under drought conditions, ABA perception and signaling regulate stomatal closure, gene expression, and stress adaptation. In both cultivars, we observed downregulation of PYR/PYL ABA receptor genes, likely indicating negative feedback regulation to prevent overactivation of ABA signaling. Increased expression of PP2C genes, which act as negative regulators in the ABA pathway, further supports this feedback loop. Meanwhile, *SnRK2* kinases showed variable expression, highlighting complex regulation by upstream signals, TFs, or cross-talk with other pathways.

In conclusion, our integrative analysis demonstrates that drought tolerance in strawberry involves a coordinated response across physiological, transcriptional, and metabolic levels. ‘Kaorino’ exhibits stronger drought tolerance than ‘Benihoppe’, likely due to superior regulation of photosynthesis, ROS detoxification, osmotic balance, and membrane stability. These traits are underpinned by the activation of ABA and MAPK signaling, lipid metabolism, and key transcription factor families.

## 5 Conclusions

‘Benihoppe’ and ‘Kaorino’ are the primary varieties of strawberries cultivated in China. In production, it has been found that ‘Kaorino’ has higher disease resistance than ‘Benihoppe’, but there is a lack of relevant research on drought resistance. In this study, through a comparative analysis under the same conditions, the physiological, biochemical, transcriptomic, and metabolomic responses of ‘Benihoppe’ and ‘Kaorino’ to drought stress were analyzed to explore their drought adaptation mechanisms and tolerance differences. The chlorophyll content decreased in both varieties under drought stress, whereas ‘Kaorino’ exhibited a smaller decline, suggesting more efficient chloroplast protection and better maintenance of photosynthesis. The activities of antioxidant enzymes (POD, SOD, and CAT) increased in both varieties, while ‘Kaorino’ showed a more significant enhancement and a smaller increase in MDA content, which demonstrated stronger oxidative stress resistance. The proline content also increased in both varieties, with ‘Kaorino’ presenting higher levels, indicating more efficient osmotic regulation. Transcriptome analysis identified 47 gene families, some of which (e.g., NAC and WRKY) are known to be involved in drought stress responses. The combined analysis of the transcriptome and metabolome revealed two key pathways associated with drought stress in strawberries: the glycerophospholipid metabolism pathway, which is related to cell membrane structure, and the MAPK signaling pathway, which is associated with environmental stress perception. The discovery of the *LPGAT* gene within the glycerophospholipid metabolism pathway may be a key factor in the differences in drought resistance observed between two varieties. In the MAPK signaling pathway, it was discovered that the *SnRK2* gene displayed a mixed expression profile, with both upregulation and downregulation, in two varieties under different drought conditions. These results will be important directions for our future work. This study has uncovered the common molecular mechanisms of strawberries under drought stress and has also identified physiological, biochemical, and molecular evidence indicating that ‘Kaorino’ exhibits superior drought resistance compared to ‘Benihoppe’. These findings provide valuable insights into the genetic and metabolic regulation of drought tolerance in strawberries, offering a foundation for breeding more drought-resistant varieties.

## Data Availability

The original contributions presented in the study are publicly available. The transcriptomic data can be found in the NCBI Sequence Read Archive (SRA) under BioProject accession PRJNA1366720. The metabolomic data are accessible in MetaboLights (https://www.ebi.ac.uk/metabolights/) under the accession number MTBLS13366.
